# Zollinger Ellison Syndrome Refractory to Medical Therapy in the Setting of Multiple Endocrine Neoplasia Type I

**DOI:** 10.7759/cureus.26468

**Published:** 2022-06-30

**Authors:** Brendan R Martino, Pedro Manibusan

**Affiliations:** 1 Internal Medicine, Tripler Army Medical Center, Honolulu, USA; 2 Gastroenterology, Tripler Army Medical Center, Honolulu, USA

**Keywords:** esophagogastroduodenoscopy (egd), endoscopic ultrasound (eus), gastrinoma, multiple endocrine neoplasia type 1 (men1), zollinger-ellison syndrome

## Abstract

Multiple Endocrine Neoplasia 1 (MEN1) syndrome is a genetic condition arising from a mutation of the MEN1 gene resulting in neuroendocrine tumor formation. Patients with MEN1 are at a higher risk of developing Zollinger-Ellison syndrome (ZES) due to the growth of neuroendocrine tumors called gastrinomas that release gastrin leading to hypersecretion of acid in the stomach resulting in severe ulcerative disease of the upper GI tract. Our case is a 42-year-old female with newly diagnosed MEN1 syndrome, presenting with acute abdominal pain and dyspepsia refractory to medical management including proton pump inhibitors (PPI) and H2 antagonists. ZES was biochemically confirmed with a secretin stimulation test and dotatate positron emission tomography/computed tomography (PET/CT) revealed multiple areas of hyper-metabolic activity within the gastrinoma triangle. However, no discrete masses could be appreciated on endoscopic ultrasound (EUS) or CT imaging that could provide a target for surgical intervention. This case elucidates not only the difficulty of gastrinoma localization in medically refractory ZES but also reinforces the need to screen patients with MEN1 presenting with acute abdominal pain and dyspepsia for ZES.

## Introduction

MEN1 syndrome is a rare autosomal dominant condition defined by the development of neuroendocrine tumors throughout the body. The most common sites involved include the parathyroid, pituitary, and pancreas [1]. The duodenum and adrenals are also commonly affected sites. Patients with MEN1 are at high risk of developing ZES due to the growth of gastrinomas leading to hypersecretion of acid. Around 90% of gastrinomas arise within the "gastrinoma triangle" which consists of the area encompassed by the head of the pancreas, cystic/common bile ducts, and the second/third portions of the duodenum [2]. About 20% of ZES cases are seen in the setting of MEN1 [3]. Roughly 85% of patients with ZES, as a consequence of MEN1, have duodenal gastrinomas [3].

## Case presentation

**Figure 1:**
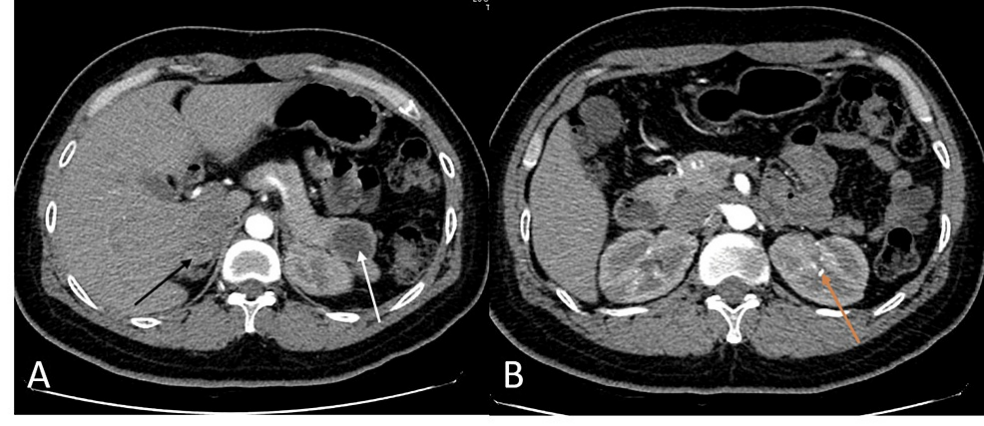
A: Axial CT showing 2.5x2.9 cm round cystic distal pancreatic mass (white arrow) and 2.0x2.7 cm oval right adrenal mass (black arrow). B: Axial CT revealing 4 mm left inter-polar renal stone (orange arrow).

**Figure 2:**
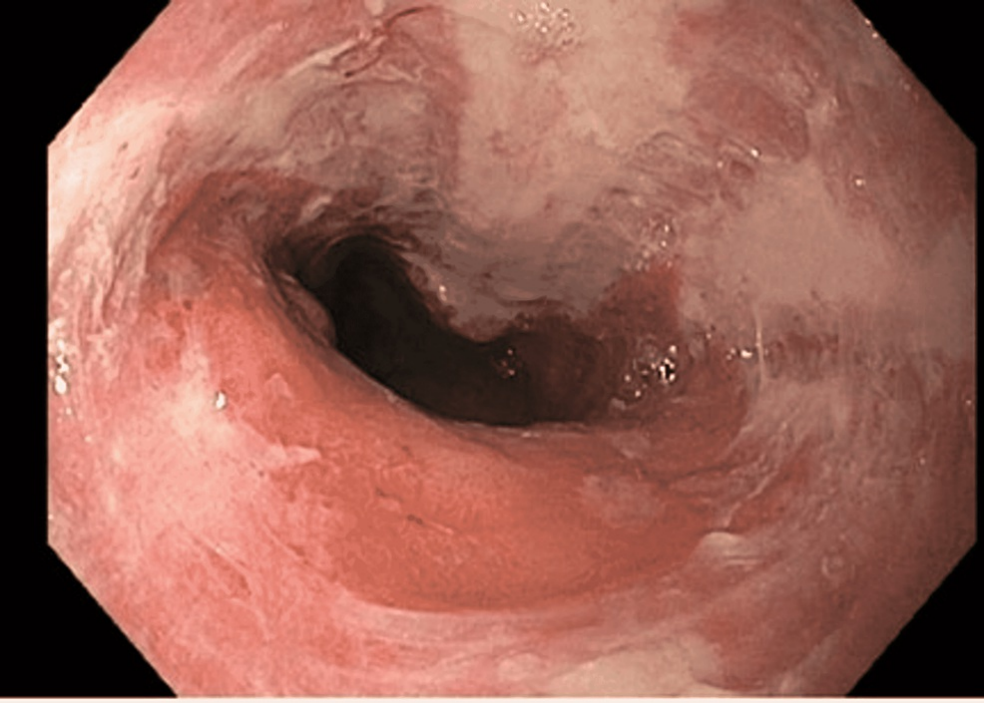
EGD view of the gastroesophageal junction with evidence for Los Angeles Grade C esophagitis EGD: esophagogastroduodenoscopy

**Figure 3:**
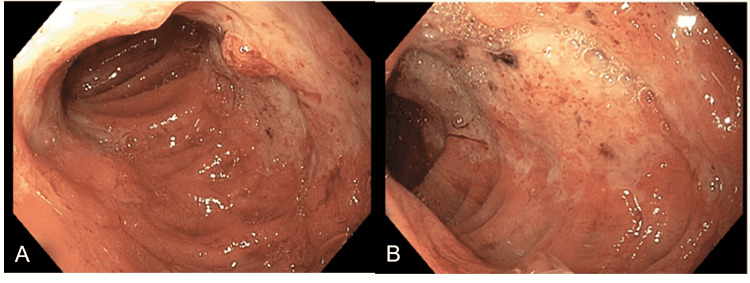
Endoscopic view of the second (A) and third (B) portions of the duodenum with diffuse duodenitis and multiple large cratered duodenal ulcers.

**Figure 4:**
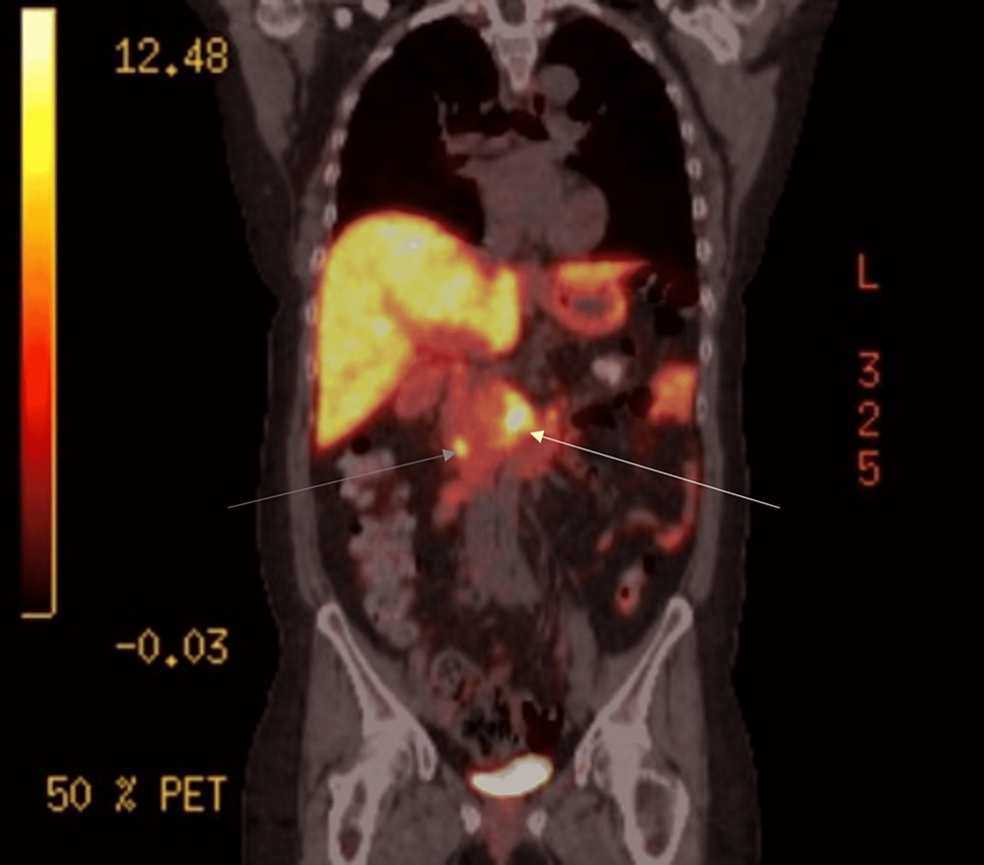
Coronal view PET/CT with hyperintensity in the second and third portions of the duodenum (blue arrow) as well as in the uncinate process of the pancreas (white arrow).

**Figure 5:**
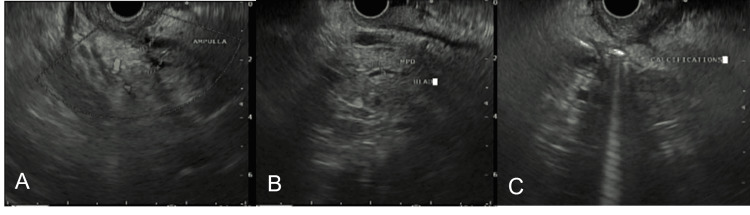
A: Ampulla view without any obvious mass lesion in the surrounding soft tissue. Common bile duct and pancreatic duct are without any evidence of abnormalities. B: Main pancreatic duct at the head of the pancreas. No evidence of mass lesion adjacent to the head of the pancreas. C: EUS image of head of the pancreas with calcification and shadowing and no obvious mass.

A 42-year-old female with a seven-month history of persistent hypercalcemia ranging between 11 to 13 mg/dl presents to the emergency department with right flank pain. CT of the abdomen and pelvis showed nephrolithiasis as well as masses in the pancreatic tail and right adrenal gland (Figure 1A, 1B). On further workup, the patient was found to have primary hyperparathyroidism with parathyroid hormone levels over 200pg/ml (reference range 10-50pg/ml). Given these constellations of findings, the patient was referred for genetic testing for suspected MEN1 syndrome and was found to have a mutation of the MEN1 gene on chromosome 11q13. The patient underwent distal pancreatectomy and right inferior parathyroidectomy. She then developed symptoms consistent with gastroesophageal reflux and was found to have a gastrin level of 220 pg/ml (reference range 30-75 pg/ml). Two month trial of H2 blockers and PPIs was initiated with very limited improvement in symptoms. Chromogranin A and pancreatic polypeptide levels were elevated to 2146 ng/ml (reference 0-101 ng/ml) and 3395 pg/ml (reference 0-418 pg/ml) respectively, raising suspicion of a gastrinoma within the head of the pancreas in the setting of her MEN1 syndrome. She was referred to GI for possible gastric outlet obstruction and was found to have diffuse esophagitis (Figure 2) with multiple gastric and duodenal ulcers (Figure 3A, 3B) on esophagogastroduodenoscopy (EGD). Dotatate PET/CT scan was obtained which showed intense uptake in the second and third portions of the duodenum as well as in the uncinate process of the pancreas with accompanying calcifications raising concern for gastrinoma (Figure 4). This was also supported by a positive secretin stimulation test (SST). Octreotide, sucralfate, and misoprostol were added to aid in symptom control. However, she developed an allergic reaction to the octreotide and was switched to lanreotide. Despite these new therapies, however, she continued to have severe pain requiring referral to pain management. The patient then underwent endoscopic ultrasound (EUS) with fine-needle aspiration (FNA) of the uncinate process which was negative for malignancy and no discrete masses were appreciated (Figure 5A-5C). Given the lack of discrete masses on imaging and negative biopsy, it was decided to perform serial EUS and CT abdomen scans every three to six months to screen for the development of a mass. Repeat EUS with biopsy of the duodenum was also scheduled given the hyperactivity on PET/CT with plans to proceed to surgical excision of the gastrinoma once identified in order to prevent metastatic spread.

## Discussion

There are two main classifications of ZES, sporadic and MEN1 associated [4]. Diagnosis involves both clinical suspicion as well as elevated gastrin and chromogranin A levels. A secretin stimulation test (SST) can also be used to confirm the diagnosis as well as to distinguish ZES from other etiologies of hypergastrinemia such as antral G-cell hyperplasia. Traditionally this would require cessation of all PPI therapy for two weeks prior to the test. However, a recent retrospective non-inferiority study evaluating SST on PPI versus SST off PPI in patients with MEN1 found that SSTs off PPI were non-inferior for sensitivity, specificity, and positive predictive value (PPV) [5]. This suggests cessation of PPI two weeks prior to SST is unnecessary in aiding in the diagnosis of ZES in patients with MEN1 syndrome with a high pretest probability of ZES. Continuing PPI could also prevent unopposed acid secretion and worsening of painful upper GI ulceration and possible perforation involved with PPI cessation in these patients.

First-line management for ZES is acid suppression therapy with PPI or H2 blockers with gastric pH goals of greater than four to prevent erosion and ulceration of the GI tract mucosa [6]. In patients refractory to these agents, prostaglandin analogs, such as misoprostol, can be added to protect the intestinal lining. In addition, many neuroendocrine tumors express somatostatin receptors, and thus there has been an investigation into the use of somatostatin analogs such as octreotide and lanreotide to reduce gastrin secretion in patients with ZES [7]. Along with suppressing acid secretion, somatostatin analogs have been found to have anti-proliferative effects on neuroendocrine tumors as demonstrated in the CLARINET trial in 2014 and was reflected in the 2020 North American Neuroendocrine Tumor Society guidelines for the medical management of advanced pancreatic neuroendocrine tumors [7,8]. These agents are particularly beneficial in patients with prior stable disease and can prolong progression-free survival.

With the emergence of acid suppression medical therapy with PPI and H2 blockers, there has been a radical reduction in the need for acid-reducing surgical interventions in patients with ZES associated with MEN1 [4]. Nevertheless, in cases refractory to medical management, such as in our patient, referral to pain management for celiac plexus block and neurolysis may provide significant aid in pain control. Ultimately, localization of the gastrinoma and surgical excision is required to reduce the acid burden and ulcerative complications in the GI tract. In patients with sporadic gastrinomas, the rates of malignancy can reach up to 90% and exploratory laparotomy should be offered, regardless of response to medical therapy to eliminate the potential for metastasis and improve survival [9]. Fortunately, surgical excision is also curative in these cases, as sporadic gastrinomas are frequently unifocal large masses (> 2 cm) as opposed to MEN1-associated gastrinomas which tend to be small multifocal masses (< 2 cm) making a surgical cure more difficult [4].

Gastrinomas can be very difficult to localize and EUS is generally recommended when surgical excision is being considered, as it is superior to CT imaging modalities in tumor localization [10]. This can be further complicated when masses are unable to be found on imaging or EUS as in this case and raise the question of a sampling error on pancreatic EUS/FNA versus normal physiologic pancreatic activity seen on Dotatate PET/CT. Given her ZES is in the setting of MEN1, there is a high suspicion of duodenal gastrinoma. Serial abdominal CT scans can be used in conjunction with EUS to help plan FNA and coordinate with pathology for bedside wet read of the biopsy specimen. 

The liver is the most common location of metastatic spread for gastrinomas, followed by the axial skeleton. Gastrinomas have high malignant potential and approximately half of all patients with ZES will have liver metastases upon initial diagnosis [6]. Metastatic spread of the gastrinoma is the main source of morbidity and mortality in these patients and thus early detection and surgical excision are of critical importance [11]. In patients with advanced neuroendocrine tumors that fail somatostatin analog therapy and are considered inoperable, there are emerging chemotherapy regimens targeting various proliferative pathways. Sunitinib, a tyrosine kinase inhibitor, has been shown to double progression-free survival in patients with inoperable advanced pancreatic neuroendocrine tumors [12]. mTOR inhibitors such as everolimus have also been studied in patients with advanced progressive pancreatic neuroendocrine tumors and found to have improved progression-free survival compared to placebo in the RADIANT-3 trial [13]. The CABINET trial is currently ongoing and evaluating the efficacy of cabozantinib in patients with advanced inoperable pancreatic and gastrointestinal neuroendocrine tumors who had previously been on everolimus. Pancreatic neuroendocrine tumors have long been considered refractory to radiation therapy. However, palliative radiotherapy in cases of unresectable pancreatic neuroendocrine tumors have been shown to have high symptom improvement and reduction in local progression and can be of great benefit in patients with advanced disease [14].

## Conclusions

MEN1 is an autosomal dominant disorder that is due to mutations in the MEN1 tumor suppressor gene resulting in neuroendocrine tumor growth throughout the body. There is a strong correlation between ZES occurring in the setting of MEN1. There are no general guidelines on the treatment and management of medically refractory ZES in the setting of MEN1 in which gastrinoma cannot be localized as surgical excision is not possible until a discrete mass is identified. Given this association, as well as the high morbidity and mortality of ZES, physicians should have a low threshold to screen for ZES in MEN1 patients complaining of gastric reflux or dyspepsia with a gastrin level, chromogranin A level, and EGD in order to allow for early diagnosis and reduce the potential of developing life-threatening complications of ZES.
